# Supplementary material for: A LAMP at the end of the tunnel: A rapid, field deployable assay for the kauri dieback pathogen, Phytophthora agathidicida
Source: PLoS One. 2020 Jan 24;15(1):e0224007. doi: 10.1371/journal.pone.0224007 (PMC6980612; doi:10.1371/journal.pone.0224007)
Supplement: S1 Table — (DOCX) [file pone.0224007.s001.docx]

**S1 Table. Sources of the oomycete mitochondrial genome sequences used when designing the *P. agathidicida* loop-mediated isothermal amplification (LAMP) assay.**

| **Species and authority** | **Family^a^** | **Genbank accession** |
| --- | --- | --- |
| *Peronospora effusa* (Grev.) Rabenh. | Peronosporaceae (15) | MH142315 |
| *Phytophthora agathidicida* B.S. Weir, Beever, Pennycook & Bellgard | Peronosporaceae (5) | MN883601 |
| *P. agathidicida* | Peronosporaceae (5) | BK011977 |
| *Phytophthora castaneae* Katsura & K. Uchida | Peronosporaceae (5) | MN883602 |
| *Phytophthora chlamydospora* Brasier & Hansen | Peronosporaceae (6) | MN883607 |
| *Phytophthora cinnamomi* Rands | Peronosporaceae (7) | BK011982 |
| *Phytophthora cocois* B.S. Weir, Beever, Pennycook, Bellgard & J.Y. Uchida | Peronosporaceae (5) | MN883603 |
| *Phytophthora colocasiae* Racib. | Peronosporaceae (2) | BK011983 |
| *Phytophthora cryptogea* Pethybr. & Laff. | Peronosporaceae (8) | BK011984 |
| *Phytophthora fallax* Dobbie & M. A. Dick | Peronosporaceae (9) | MN883608 |
| *Phytophthora heveae* A.W. Thomps. | Peronosporaceae (5) | MN883604 |
| *Phytophthora infestans* (Mont.) de Bary | Peronosporaceae (1) | NC_014280 |
| *Phytophthora kernoviae* Brasier | Peronosporaceae (10) | BK011986 |
| *Phytophthora* *litchii* (C.C. Chen ex W.H. Ko, H.S. Chang, H.J. Su, C.C. Chen & L.S. Leu) Voglmayr, Göker, Riethm. & Oberw. | Peronosporaceae (4) | BK011980 |
| *Phytophthora multivora* P.M. Scott & T. Jung | Peronosporaceae (2) | BK011988 |
| *Phytophthora nicotianae* Breda de Haan | Peronosporaceae (1) | BK011989 |
| *Phytophthora palmivora* (E.J. Butler) E.J. Butler | Peronosporaceae (4) | MN883609 |
| *Phytophthora pinifolia* Alv. Durán, Gryzenh. & M.J. Wingf. | Peronosporaceae (6) | BK011991 |
| *Phytophthora pluvialis* Reeser, Sutton & Hansen | Peronosporaceae (3) | BK011992 |
| *Phytophthora polonica* Belbahri et al. | Peronosporaceae (9) | NC_029397 |
| *Phytophthora ramorum* Werres, De Cock & Man in 't Veld | Peronosporaceae (8) | NC_009384 |
| *Phytophthora sojae* Kaufm. & Gerd*.* | Peronosporaceae (7) | NC_009385 |
| *Phytophthora* taxon totara | Peronosporaceae (15) | BK011993 |
| *Phytophthora tropicalis* Aragaki & J. Y. Uchida | Peronosporaceae (3) | BK011981 |
| *Plasmopara viticola* (Berk. & M.A. Curtis) Berl. & De Toni | Peronosporaceae (16) | KY885002 |
| *Pythium ultimum* Trow | Pythiaceae | NC_014280 |

^a^For Peronosporaceae the clade numbers of Bourret et al. (2018) are also provided.
