# Supplementary material for: A LAMP at the end of the tunnel: A rapid, field deployable assay for the kauri dieback pathogen, Phytophthora agathidicida
Source: PLoS One. 2020 Jan 24;15(1):e0224007. doi: 10.1371/journal.pone.0224007 (PMC6980612; doi:10.1371/journal.pone.0224007)
Supplement: S2 Table — (DOCX) [file pone.0224007.s002.docx]

**S2 Table. Location details for** **field collected soil samples used in comparisons of extended bioassay and *P. agathidicida* loop-mediated isothermal assay.**

| **Accession no.^a^** | **Collection location** | **GPS coordinates^b^** |
| --- | --- | --- |
| Waitākere Ranges Regional Park | | |
| HTHF1003 | Maungaroa Ridge | -36.946, 174.483 |
| HTHF1014 | Maungaroa Ridge | -36.947, 174.481 |
| HTHF1018 | vicinity of Lower Kauri Track | -36.888, 174.515 |
| HTHF1020 | vicinity of Lower Kauri Track | -36.888, 174.515 |
| HTHF1035 | vicinity of Huia Dam | -36.998, 174.563 |
| HTHF1037 | vicinity of Huia Dam | -36.998, 174.563 |
| Waipoua Forest Sanctuary | | |
| HTHF1043 | State Highway 12 | -35.646, 173.558 |
| HTHF1054 | State Highway 12 | -35.645, 173.558 |
| HTHF1055 | State Highway 12 | -35.646, 173.558 |
| HTHF1071 | State Highway 12 | -35.649, 173.563 |
| HTHF1072 | State Highway 12 | -35.650, 173.563 |
| HTHF1081 | State Highway 12 | -35.650, 173.562 |
| HTHF1083 | State Highway 12 | -35.650, 173.562 |
| HTHF1090 | Waipoua River Bridge | -35.652, 173.570 |
| HTHF1091 | Waipoua River Bridge | -35.652, 173.570 |

^a^Samples drawn from a wider set of collected as part of the “Health Trees Health Forests” programme.

^b^Coordinate system is World Geodetic System 1984 (WGS84).
