# Supplementary material for: A LAMP at the end of the tunnel: A rapid, field deployable assay for the kauri dieback pathogen, Phytophthora agathidicida
Source: PLoS One. 2020 Jan 24;15(1):e0224007. doi: 10.1371/journal.pone.0224007 (PMC6980612; doi:10.1371/journal.pone.0224007)
Supplement: S3 Table — (DOCX) [file pone.0224007.s003.docx]

**S3 Table. Comparison of the *Phytophthora agathidicida* LAMP assay** **target sequence and the corresponding locus in other Oomycetes.**

| **Species and authority** | **Family^a^** | **Results of comparison^b^** | **Genbank accessions** |
| --- | --- | --- | --- |
| *Achlya hypogyna* Coker & Pemberton | Saprolegniaceae | 100, 86.8 | KF226724 |
| *Albugo laibachii* Thines & Y.J.Choi | Albuginaceae | 100, 87.3 | FR826008 |
| *Aphanomyces astaci* Schikora | Saprolegniaceae | 100, 86.4 | KX405004 |
| *Aphanomyces invadans* David & Kirk | Saprolegniaceae | 100, 86.4 | KX405005 |
| *Bremia lactucae* Regel | Peronosporaceae (16) | 100, 86.8 | FJ810099-FJ810101, NC_040179 |
| *Globisporangium paroecandrum* (Drechsler) Uzuhashi, Tojo & Kakish. | Pythiaceae | 100, 88.6-89.0 | MG020413, MG020415 |
| *Globisporangium sylvaticum* (W.A. Campb. & F.F. Hendrix) Uzuhashi, Tojo & Kakish. | Pythiaceae | 100, 89.5-89.9 | MG020395-MG020397 |
| *Hyaloperonospora arabidopsidis* (Gäum.) Göker, Riethm., Voglmayr, Weiß & Oberw. | Peronosporaceae (15) | 100, 93.0 | BK011976 |
| *Peronospora belbahrii* Thines | Peronosporaceae (15) | 100, 91.7 | BK011978 |
| *Peronospora effusa* (Grev.) Rabenh. | Peronosporaceae (15) | 100, 93.4 | MH142315, MH325167 |
| *Peronospora hyoscyami* de Bary f.sp. *tabacina* (Adam) Skalicky | Peronosporaceae (15) | 100, 93.9 | KT893455, KT893456 |
| *Peronospora sparsa* Berk. | Peronosporaceae (15)^e^ | 100, 93.0 | DQ887772 |
| *Phytophthora* × *alni* Brasier & S.A.Kirk | Peronosporaceae (7) | 100, 93.4 | FJ810084 |
| *Phytophthora andina* Adler & Flier | Peronosporaceae (1) | 100, 92.5 | HM590419, KJ408269 |
| *Phytophthora cactorum* (Lebert & Cohn) J. Schröt. | Peronosporaceae (1) | 100, 94.7 | BK011979 |
| *Phytophthora* × *cambivora* (Petri) Buisman | Peronosporaceae (7) | 100, 93.4 | FJ810086 |
| *Phytophthora capitosa* M. A. Dick & Dobbie | Peronosporaceae (9) | 100, 92.1 | MN883606 |
| *Phytophthora capsici* Leonian | Peronosporaceae (2) | 100, 92.1 | MG570037 |
| *Phytophthora castaneae* Katsura & K. Uchida | Peronosporaceae (5) | 100, 96.5 | MN883602 |
| *Phytophthora chlamydospora* Brasier & Hansen | Peronosporaceae (6) | 100, 92.5 | MN883607 |
| *Phytophthora cinnamomi* Rands | Peronosporaceae (7) | 100, 93.0 | FJ810088 |
| *Phytophthora citrophthora* | Peronosporaceae (2) | 100, 91.7 | FJ810091 |
| *Phytophthora cocois* B.S. Weir, Beever, Pennycook, Bellgard & J.Y. Uchida | Peronosporaceae (5) | 100, 96.0 | MN883603 |
| *Phytophthora colocasiae* Racib. | Peronosporaceae (2) | 100, 92.1 | BK011983 |
| *Phytophthora cryptogea* Pethybr. & Laff. | Peronosporaceae (8) | 100, 93.0 | BK011984 |
| *Phytophthora fallax* Dobbie & M. A. Dick | Peronosporaceae (9) | 100, 93.9 | MN883608 |
| *Phytophthora fragariae* Hickman | Peronosporaceae (7) | 100, 91.7 | BK011985 |
| *Phytophthora gonapodyides* (H.E. Petersen) Buisman | Peronosporaceae (6) | 100, 92.5 | FJ810093 |
| *Phytophthora heveae* A.W. Thomps. | Peronosporaceae (5) | 100, 95.6 | MN883604 |
| *Phytophthora humicola* W.H. Ko & Ann | Peronosporaceae (6) | 100, 93.0 | FJ810094 |
| *Phytophthora infestans* (Mont.) de Bary | Peronosporaceae (1) | 100, 92.1-92.5 | AY894835, AY898627, AY898628, U17009, FJ810095, MH286884-MH286887 |
| *Phytophthora inundata* Brasier, Sánchez-Hernandez & S. A. Kirk | Peronosporaceae (6) | 100, 92.1 | FJ810090 |
| *Phytophthora ipomoeae* Flier & Grünwald | Peronosporaceae (1) | 100, 91.7 | HM590420 |
| *Phytophthora kernoviae* Brasier | Peronosporaceae (10) | 100, 92.1 | BK011986 |
| *Phytophthora lateralis* Tucker & Milbrath | Peronosporaceae (8) | 100, 92.5 | BK011987 |
| *Phytophthora* *litchii* (C.C. Chen ex W.H. Ko, H.S. Chang, H.J. Su, C.C. Chen & L.S. Leu) Voglmayr, Göker, Riethm. & Oberw. | Peronosporaceae (4) | 100, 93.9 | BK011980 |
| *Phytophthora megasperma* Drechsler | Peronosporaceae (6) | 100, 93.0 | L16863 |
| *Phytophthora mirabilis* Galindo & H.R. Hohl | Peronosporaceae (1) | 100, 92.5 | HM590421 |
| *Phytophthora multivora* P.M. Scott & T. Jung | Peronosporaceae (2) | 100, 93.9 | BK011988 |
| *Phytophthora nicotianae* Breda de Haan | Peronosporaceae (1) | 100, 91.7-92.5 | FJ810089, KY851301 |
| *Phytophthora* sp. *novaeguineae* | Peronosporaceae (5) | 100, 96.0 | MN883605 |
| *Phytophthora palmivora* (E.J. Butler) E.J. Butler | Peronosporaceae (4) | 100, 92.5 | FJ810092 |
| *Phytophthora parasitica* Dastur | Peronosporaceae (1) | 100, 92.5 | BK011990 |
| *Phytophthora phaseoli* Thaxter | Peronosporaceae (1) | 100, 92.1 | HM590418 |
| *Phytophthora pinifolia* Alv. Durán, Gryzenh. & M.J. Wingf. | Peronosporaceae (6) | 100, 90.8 | BK011991 |
| *Phytophthora pluvialis* Reeser, Sutton & Hansen | Peronosporaceae (3) | 100, 93.7 | BK011992 |
| *Phytophthora polonica* Belbahri et al. | Peronosporaceae (9) | 100, 91.7 | KT946598 |
| *Phytophthora ramorum* Werres, De Cock & Man in 't Veld | Peronosporaceae (8) | 100, 92.5 | DQ832718, EU427470 |
| *Phytophthora rubi* (W.F. Wilcox & J.M. Duncan) W.A. Man in 't Veld | Peronosporaceae (7) | 100, 93.9 | FJ810087 |
| *Phytophthora sansomeana* E.M. Hansen & Reeser | Peronosporaceae (8) | 100, 91.7 | NC_045089 |
| *Phytophthora sojae* Kaufm. & Gerd*.* | Peronosporaceae (7) | 100, 91.7 | DQ832717 |
| *Phytophthora* taxon totara | Peronosporaceae (15) | 100, 94.3 | BK011993 |
| *Phytophthora tropicalis* Aragaki & J. Y. Uchida | Peronosporaceae (3) | 100, 91.7 | BK011981 |
| *Plasmopara halstedii* (Farl.) Berl. & De Toni | Peronosporaceae (16) | 100, 90.4 | FJ810096-FJ810098 |
| *Plasmopara viticola* (Berk. & M.A. Curtis) Berl. & De Toni | Peronosporaceae (16) | 100, 86.8-89.9 | AY696297, DQ209286, DQ459459- DQ459469, KF652186, KF652188-KF652195, KF652197, KY403517- KY403519, KY403527, MF102263, MG757168 |
| *Pseudoperonospora cubensis* (Berkeley & Curtis) Rostovtsev | Peronosporaceae (15) | 100, 92.5 | KT072718 |
| *Pseudoperonospora humuli* (Miyabe & Takah.) G.W. Wilson | Peronosporaceae (15)^e^ | 100, 93.0 | NC_042478 |
| *Pythium aphanidermatum* (Edson) Fitzp. | Pythiaceae | 100, 89.0 | MG020387-MG020388 |
| *Pythium heterothallicum* W.A. Campb. & F.F. Hendrix | Pythiaceae | 100, 90.8 | KJ639169 |
| *Pythium insidiosum* De Cock, L.Mend., A.A.Padhye, Ajello & Kaufman | Pythiaceae | 100, 89.0 | AP014838 |
| *Pythium irregulare* Buisman | Pythiaceae | 100, 89.0 | MG020401 |
| *Pythium splendens* Hans Braun | Pythiaceae | 100, 89.5 | KJ639171 |
| *Pythium ultimum* Trow | Pythiaceae | 100, 89.5-91.2 | GU138662-GU138663, KJ639140-KJ639141, KJ639143-KJ639144, KJ639146-KJ639150, KJ639152, KJ639157, KJ639160, KJ639167, MG020403, MG020410 |
| *Saprolegnia ferax* (Gruith.) Kütz. | Saprolegniaceae | 100, 86.8 | AY534144 |
| *Thraustotheca clavata* (De Bary) Humphrey | Saprolegniaceae | 100, 85.1 | KF226725 |

^a^For Peronosporaceae the clade numbers of Bourret et al. (2018) are also provided.

^b^The percent coverage of the 227 nucleotide long assay target sequence and percent identity with the *Phytophthora agathidicida* sequence.
