# Supplementary material for: A LAMP at the end of the tunnel: A rapid, field deployable assay for the kauri dieback pathogen, Phytophthora agathidicida
Source: PLoS One. 2020 Jan 24;15(1):e0224007. doi: 10.1371/journal.pone.0224007 (PMC6980612; doi:10.1371/journal.pone.0224007)
Supplement: S1 Fig — (PDF) [file pone.0224007.s004.pdf]

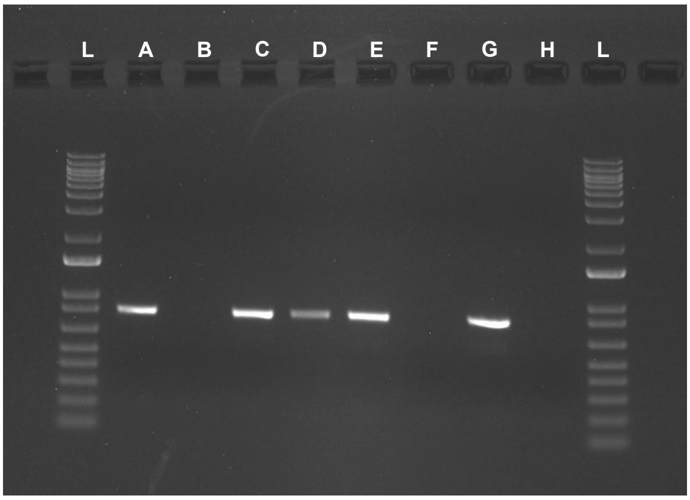

S4 Figure. Endpoint visualisation of PCR amplification products using SYBR Safe (Invitrogen) following electrophoresis on a 1% TAE agarose gel. Lane A, 5 ng total bait DNA from Waitakere Ranges Regional Park sample HTHF 1003; lane B, 5 ng total bait DNA from Waitakere Ranges Regional Park sample HTHF 1018; lane C, 5 ng total bait DNA from Waitakere Ranges Regional Park sample HTHF 1035; lane D, 5 ng total bait DNA from Waipoua Forest Sanctuary sample HTHF 1033; lane E, 5 ng total bait DNA from Waipoua Forest Sanctuary sample HTHF 1081; lane F, 5 ng total bait DNA from Waipoua Forest Sanctuary sample HTHF 1090; lane G, 2 pg total DNA isolate ICMP18244; lane H, no DNA control; lane L, 1 kb plus DNA ladder.
