# Supplementary material for: A LAMP at the end of the tunnel: A rapid, field deployable assay for the kauri dieback pathogen, Phytophthora agathidicida
Source: PLoS One. 2020 Jan 24;15(1):e0224007. doi: 10.1371/journal.pone.0224007 (PMC6980612; doi:10.1371/journal.pone.0224007)
Supplement: S1 Raw images — (PDF) [file pone.0224007.s005.pdf]

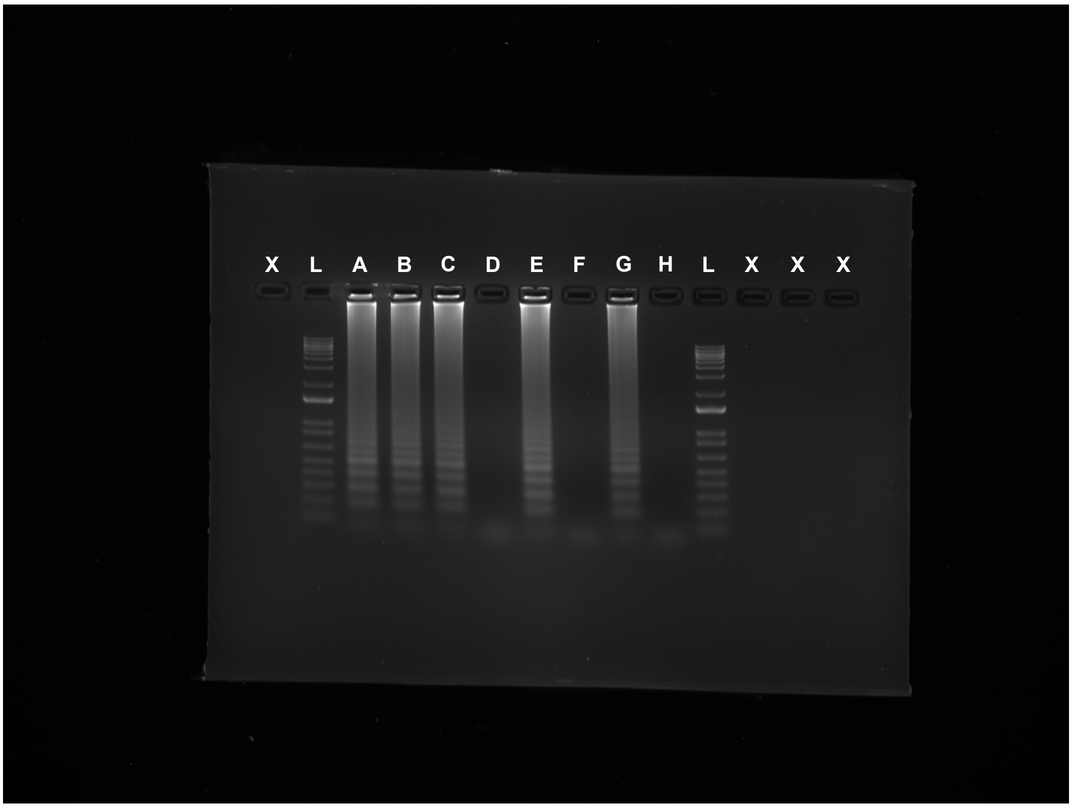

Original gel image supporting Figure 3. Endpoint visualisation of LAMP products using SYBR Safe following electrophoresis on a 1% TAE agarose gel. Lane A, 2 pg PCR amplification products from ICMP 18244; lane B, 2 pg total DNA from isolate ICMP 18244; lane C, 2 pg total DNA from isolate ICMP 18210; lane D, 5 ng total bait DNA from Waitakere Ranges Regional Park sample HTHF 1018; lane E, 5 ng total bait DNA from Waitakere Ranges Regional Park sample HTHF 1020; lane F, 5 ng total bait DNA from Waipoua Forest Sanctuary sample HTHF 1072; lane G, 5 ng total bait DNA from Waipoua Forest Sanctuary sample HTHF 1081; lane H, no DNA control; lane L, 1 Kb plus DNA ladder; lane X, lanes not loaded (only the two lanes closest to 1 Kb plus ladder are visible in final figure).

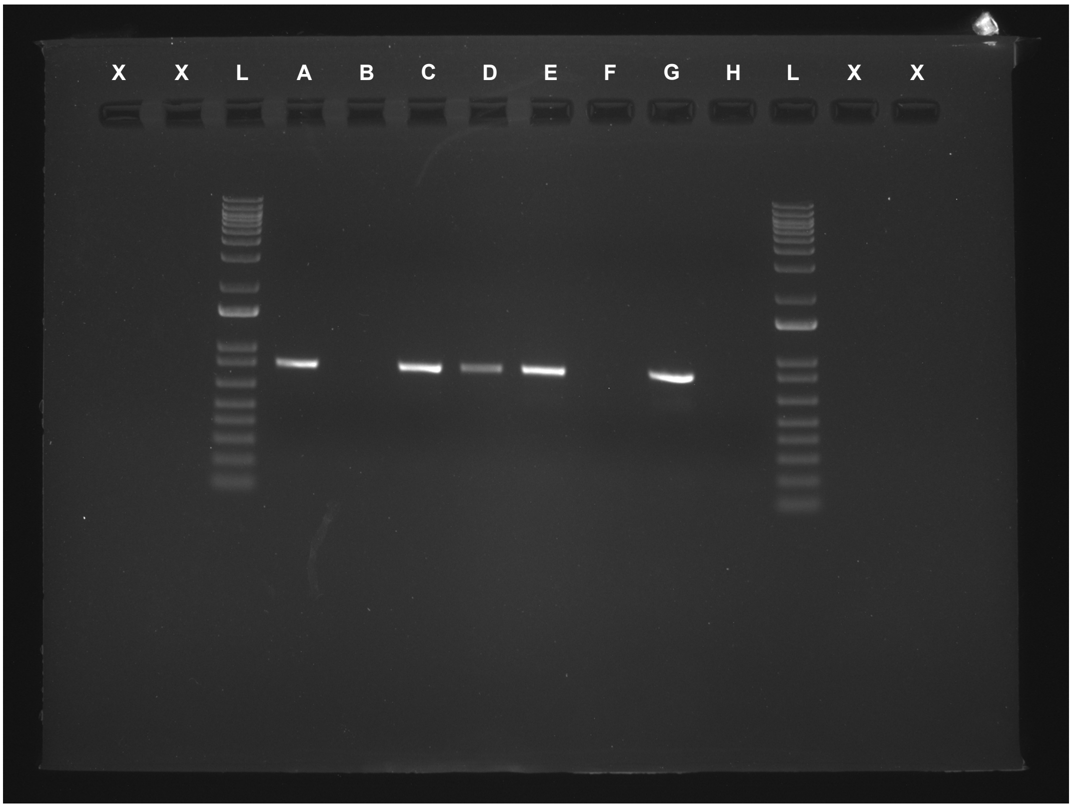

Original gel image supporting S4 Figure. Endpoint visualisation of PCR amplification products using SYBR Safe following electrophoresis on a 1% TAE agarose gel. Lane A, 5 ng total bait DNA from Waitakere Ranges Regional Park sample HTHF 1003; lane B, 5 ng total bait DNA from Waitakere Ranges Regional Park sample HTHF 1018; lane C, 5 ng total bait DNA from Waitakere Ranges Regional Park sample HTHF 1035; lane D, 5 ng total bait DNA from Waipoua Forest Sanctuary sample HTHF 1033; lane E, 5 ng total bait DNA from Waipoua Forest Sanctuary sample HTHF 1081; lane F, 5 ng total bait DNA from Waipoua Forest Sanctuary sample HTHF 1090; lane G, 2 pg total DNA from isolate 18244; lane H, no DNA control; lane L, 1 Kb plus DNA ladder; lane X, lanes not loaded (only the two lanes closest to 1 Kb plus ladder are visible in final figure).
